# DNA sampling from eggshells and microsatellite genotyping in rare tropical birds: Case study on Brazilian Merganser

**DOI:** 10.1590/1678-4685-GMB-2016-0297

**Published:** 2017-10-02

**Authors:** Thais Augusta Maia, Sibelle Torres Vilaça, Luciana Resende da Silva, Fabricio Rodrigues Santos, Gisele Pires de Mendonça Dantas

**Affiliations:** 1 Laboratório de Ecologia e Evolução de Aves, Programa de Pós-Graduação em Biologia de Vertebrados, Pontifícia Universidade Católica de Minas Gerais, Belo Horizonte, MG, Brazil; 2 Department Evolutionary Genetics, Leibniz Institute for Zoo and Wildlife Research, and Berlin Center for Genomics in Biodiversity Research, Berlin, Germany; 3 Laboratório de Biodiversidade e Evolução Molecular, Instituto de Ciências Biológicas, Universidade Federal de Minas Gerais, Belo Horizonte, MG, Brazil

**Keywords:** DNA, noninvasive samples, genetic diversity, endangered birds, Mergus

## Abstract

This study shows that sampling maternal DNA from hatched and abandoned eggshells is a viable noninvasive strategy for studying the genetics of rare or endangered tropical birds, as exemplified here by the Brazilian Merganser (*Mergus octosetaceus*). Eighteen microsatellites were isolated from enriched libraries and nine heterologous loci from related species were tested. Seven loci were amplified successfully, with five of them being polymorphic. These loci exhibited amplicons ranging from 110 to 254 bp for 132 samples, with 60 from eggshells and 72 from blood or muscle samples. The number of alleles for *M. octosetaceus* ranged from one to six (mean = 3.71), which is low compared to *M. merganser* (1-15 alleles), a ‘least concern’ species. Genetic diversity did not differ significantly between noninvasive and invasive samples (Z(u) = 0.31, *p* = 0.37). Thus, noninvasive sampling, as demonstrated here with eggshells, provides an efficient means to assess genetic diversity in tropical birds without the need to capture and handle them.

Noninvasive DNA sampling has become an important method for collecting valuable genetic data for the management and conservation of rare and endangered species (Waits and Paetkau, 2005; Beja-Pereira *et al.*, 2009; Egloff *et al.*, 2009). For birds, the use of eggshells and feathers are good sources of biological samples (Pearce *et al.*, 1997; Bush *et al.*, 2005; Egloff *et al.*, 2009). However, noninvasive sampling from tropical species is particularly challenging due to several environmental factors, including high temperatures, precipitation and UV radiation, which can promote DNA degradation (Brinkman *et al.*, 2010; Vynne *et al.*, 2012; Wultsch *et al.*, 2014).

Some studies have used microsatellites for accessing genetic diversity from noninvasive samples (Presti *et al.*, 2013; Moodley *et al.*, 2015), due to the fact that these markers are widely distributed in the genome, have high levels of polymorphism, and are easy to detect using PCR (Hoshino *et al.*, 2012). Thus, microsatellites have been frequently used to monitor biodiversity, evaluate paternity and to attribute individuals to populations (Beebee and Rowe, 2008). Furthermore, primers developed in one species may be used in related taxa (Barbará *et al.*, 2007; Santos *et al.*, 2012). Nonetheless the success of cross-species amplification of microsatellites depends on conservation of flanking regions, the polymorphism level, the phylogenetic relatedness among the species involved, with success being higher in closely related species (Barbará *et al.*, 2007; Presti *et al.*, 2013; Moodley *et al.*, 2015). In this context, the use of noninvasive samples and polymorphic markers, such as microsatellites, can help to monitor the genetics of rare and endangered species.

The Brazilian Merganser (*Mergus octosetaceus*) is considered critically endangered, with a global population estimated to be less than 250 individuals, distributed among three or four disjointed populations located in protected areas of Brazil (BirdLife International, 2015). Until the moment, only one genetic study was conducted with this species, where two mitochondrial lineages were observed, with high divergence and low variability within each (Vilaça *et al.*, 2012). Thus, questions were raised about the genetic diversity and evolutionary history of the Brazilian Merganser.

The present study aims to: 1) test a noninvasive method of sampling avian DNA from abandoned eggs and hatched eggshells, 2) develop new microsatellite markers for the Brazilian Merganser through sequencing of enriched libraries, and 3) test cross-amplification using previously described primers for other species of Anseriformes.

In total, 132 Brazilian Merganser samples were collected in the Serra da Canastra National Park over the course of eight years (2005-2013), including 59 blood tissue samples from captured individuals, 13 muscle samples from dead individuals, and 60 noninvasive samples from eggshells (membrane and shells) of abandoned or hatched eggs in nests. The nests were monitoring through the entire study period, adults were ringed and only one eggshell of each nest was used, trying to avoid resampling. The length of time that the eggshell was exposed to environmental conditions could not be determined. All samples were georeferenced, kept in plastic bags, and stored in a refrigerator for subsequent analysis. Genomic DNA was extracted from eggshell (shell and intern membrane), blood and muscle samples using the protocol described in Sambrook *et al.* (2001). They were treated with lysis solution (TrisHCl 0.1 M, pH 8.0; EDTA 0.5 M; SDS 0.2%; NaCl 1 M) containing proteinase K, overnight at 37 °C, followed one wash with phenol:chloroform:isoamyl alcohol (25:24:1) and one wash with chloroform:isoamyl alcohol (24:1), precipitation with isopropanol 100% and ethanol 70%. The DNA concentration of all samples was quantified spectophotometrically using NanoDrop ND-1000 (Nanodrop, USA).

Microsatellite enriched libraries were constructed following the methodology described in Billotte *et al.* (1999), using streptavidin magnetic-coated beads (Promega, Madison, WI). DNA (~5 μg) extracted from one blood sample was digested with the enzyme *Rsa*I to create an appropriately sized genomic library (400-1000 bp). The digested fragments were ligated to the adaptors RSA21 (5′ CTCTTGCTTACGCGTGGACTA 3′) and RSA25 (5′ TAGTCCACGCGTAAGCAAGAGCACA 3′). The DNA library was enriched for dinucleotide sequences using (CT)_8_ and (GT)_8_ biotinylated probes that were bound to streptavidin magnetic-coated beads (Streptavidin MagneSphere Paramagnetic Particles, Promega). Selected fragments were amplified by PCR using primer sequences complementary to the RSA21 adapter and then cloned into the pGEM-T vector (Promega). Competent XL1-blue *E. coli* cells were transformed with the recombinant plasmids and cultivated on agar medium containing ampicillin and 100 μg/mL of X-galactosidase. Single white colonies were transferred onto microplates for long-term storage at −80 °C. Ninety-six clones containing an insert were sequenced using a MEGABACE 1000 automated sequencer (GE Healthcare Life Sciences). Microsatellites were identified using the Simple Sequence Repeat Identification Tool (Temnykh *et al.*, 2001).

Primers were designed for 18 loci containing microsatellites using Primer3Plus software (Untergasser *et al.*, 2007). An M13 tail was added to the forward primer of each primer pair to allow fluorescent labeling during amplification reactions (Schuelke, 2000). In addition, we tested the cross-amplification of additional microsatellite loci using four primer pairs developed for *M. merganser* (Gautschi and Koller, 2005), four primer pairs developed for *Anas platyrhynchus* (Maak *et al.*, 2003), and one primer pair developed for Anatidae (Buchholz *et al.*, 1998) (Supplementary material Table S1).

PCR amplifications were performed in a 10 μL reaction volume containing 25 ng of genomic DNA, 0.7 μM forward primer, 8 μM reverse primer, 8 μM M13tag marked with HEX or FAM, 250 μM dNTPs, 0.5 U *Taq* DNA polymerase (Lifetech Carlsbad, EUA) and 1X reaction buffer (20 mM TrisCl, 0.1 mM EDTA, 1 mM DTT) with a final concentration of 2.5 mM MgCl_2_. We used a touchdown PCR program of 95 °C for 15 min, followed by 30 cycles at 94 °C for 30 s, 65 °C for 30 s, reducing 0.5 °C per cycle, and 72 °C for 1 min. The next 20 cycles were 94 °C for 30 s, 48 °C per 30 s, and 72 °C for 1 min. The last cycle was followed by a 30 min extension step at 72 °C. Two parameters were used to consider unsuccessful amplifications after several attempts at standardization: a locus did not amplify, or the locus showed unspecific bands. We tested each microsatellite locus with different PCR conditions that varied in concentrations of MgCl_2_ (1.0 mM to 5.0 mM), temperature gradient, and DNA concentration. The amplicons were diluted, mixed with internal size standard Rox500 (Applied Biosystems, Foster City, CA, USA), run on an ABI3130 instrument (Applied Biosystems), and analyzed using GeneMapper (Applied Biosystems). Genotyping of the samples was performed at least twice to confirm the amplicons. When the sample showed same genotype in two independent runs we considered this to the correct genotyping. When the sample showed distinct genotypes in two first runs we did another run (third run) and determined the genotype by consensus. The consensus decision was done by eye, and the error rate was very low (< 2%), with the genotyping confirmed either by the second or third run.

MICRO-CHECKER was used to evaluate dropout and null alleles in the invasive and noninvasive samples (Van Oosterhout *et al.*, 2004). Loci linkage loci was test in GenePop (Raymond and Rousset, 1995). Allelic richness, expected and observed heterozygosity, exact tests for departure from Hardy–Weinberg equilibrium and coefficients of inbreeding (FIS) were determined using GeneAlEx v6.5 (Peakall and Smouse, 2012).

DNA was successfully extracted from eggshells exposed to environmental conditions before sampling, in spite of the DNA concentration of noninvasive samples being lower (~30 ng/μl) than that of invasive samples (> 300 ng/μl). Most avian genetics studies that employ noninvasive sampling use feathers (Segelbacher, 2002; Presti *et al.*, 2013), although a few have used eggshell membranes (Trimbos *et al.*, 2009), eggshell powder by surface abrasion (Egloff *et al.*, 2009), or eggshell swabbing (Martin-Galvez *et al.*, 2011).

Of the 27 microsatellite loci tested (Table S1), amplification occurred in seven (MOCC3, MOCD4, MOCF12, MOCH3, MOCH5, Aph08, MM03), with amplicons ranging from 110 to 254 bp (Table 1). MOCF12 (allele 194) and MM03 (allele 231) were found to be monomorphic. The loci Aph08 and MM03 were sequenced to confirm the microsatellites. In the pairwise locus analyses, none showed a linkage disequilibrium. Sixty-five percent of the invasive samples and 50 percent of the noninvasive samples were successfully amplified for seven loci, of which five exhibited polymorphisms with two to six alleles. Our results showed lower amplification success for heterologous markers (22.2%) *vs.* species-specific loci (27.7%). Mutations in flanking regions or disruptions within tandem-repeated elements may lead to failure of amplification and reduced levels of polymorphism in cross amplification (Moodley *et al.*, 2015). Accordingly, only one heterologous marker showed success of amplification in Brazilian Merganser, therefore development of specific markers allowed access genetic diversity with success. These markers will allow to assess genetic diversity in wild populations and in distinct kinds of samples.

**Table 1:** Comparison of invasive and noninvasive samples for microsatellites with successful amplification in the Brazilian Merganser: N: number of samples; Na: number of alleles; H_o_: observed heterozygosity; H_e_: expected heterozygosity; HWE, FIS: Inbreeding coefficient; HWE test p-value < 0.05 was represented in bold. (1) loci developed in this study (2) locus from Maak *et al.* (2003); sequence of M13 tail: TTTTCCCAGTCACG.

					Noninvasive Samples					Invasive samples					
Loci	Primers	Repeat motif	Range alleles	Null alleles	N	Na	Ho	He	HWE *p*-value	N	Na	Ho	He	HWE *p*-value	FIS
**MOCC3** ^1^	CAGGGCAACAAAATCAGGTTC	(TG)_10_			45	2	0.244	0.401	**< 0.01**	28	2	0.429	0.459	0.69	0.223
	CCAAGTGAGAAACAAAACC		218-220	0.07											
**MOCD4** ^1^	CGCGCTAGCTGTAAGGCTCAT	(AC)_8_			45	6	0.089	0.495	**< 0.01**	41	4	0.049	0.224	**< 0.01**	0.809
	CCAGAAAAGCCTGTGTTGGT		176-206	0.32											
**MOCH3** ^1^	CAGCTGCAGTCTGTGGGAGAT	(CA)_2_TG(CA)_7_			46	3	0.022	0.104	**< 0.01**	34	5	0.059	0.167	**< 0.01**	0.704
	CAGTGCAAAAGAGGGCAGAG		234-254	0.00											
**MOCH5** ^1^	CCAATGGGGTCATTGTTGAGA	(CA)_7_TACATA(CA)_5_			56	3	0.036	0.275	**< 0.01**	37	3	0.054	0.237	**< 0.01**	0.825
	GCATTGTATTTACAGAGG		196-204	0.26											
**APH08** ^2^	AAAGCCCTGTGAAGCGA	(CA)_12_			37	4	0.081	0.469	**< 0.01**	22	2	0.136	0.201	0.14	0.669
	TGTGTGTGCATCTGGGTGTGT		110-118	0.12											
**MOCF12**	CAAAGCTTCCTCCCTCACTCC	(GT)_9_	194	–		1	–	–	–		1	–	–	–	–
	AGCTTCCTGTGGCATAGCAT														
**MM3**	AAGTACATGTAAAAGCTGAAGTTGC	(CA)_16_	231	–		1	–	–	–		1	–	–	–	–
	TTGCCTGATAAAAGGAATGC														
		**Overall**			72	2.85 ±2.19	0.067	0.249		2.57 ±1.52	0.104	0.184		0.652	

Previous studies with noninvasive samples found that these can result in low-quality DNA that may even be contaminated with PCR inhibitors (Horváth *et al.*, 2005; Presti *et al.*, 2013), thus hindering genotyping (Taberlet *et al.*, 1996; Mukesh *et al.*, 2011). We minimized the possibility of genotyping errors by running each sample at least twice and by testing the occurrence of allele dropout in MICRO-CHECKER. No sign of allele dropout was detected in any of the loci. However, it showed the possibility of null alleles for some loci (Table 1) due to an excess of homozygotes, where all loci from noninvasive samples showed an HWE bias, as well as three loci from invasive samples. This excess of homozygotes could also be consequence of inbreeding, given that the population is small and apparently isolated (FIS 0.66).

Genetic diversity (Table 1) did not differ significantly between invasive and noninvasive samples (Mann-Whitney Z(u) = 0.31, p = 0.37), with mean allelic number 2.51 (±1.52) and 2.85 (±2.19), and observed heterozygosity of 0.067 and 0.104 for noninvasive and invasive samples, respectively (Table 1). The Brazilian Merganser showed low allelic richness (1 to 6 alleles; mean = 3.71) when compared to its sister species, the least concern *M. merganser* (1 to 15 alleles per locus, mean = 6.88); Gautschi and Koller 2005), with the *caveat* that the panel used in these studies did not include the same loci.

In conclusion, the results of the present study should encourage the practice of extracting maternal DNA from eggshells found in the field, even if deposited several days before sampling. However, the panel of microsatellite markers used in this study showed some limitations, such as low polymorphisms and successful of amplification in only about 50% of the samples. Despite these limitations, the microsatellites revealed low genetic diversity in the critically endangered Brazilian Merganser. Furthermore, these new markers should allow to assess the genetic diversity in other wild populations with higher samples size, and consequently will be of help in management and protection decisions.

## Supplementary Material

The following online material is available for this article:

Table S1 : The 27 loci tested for amplification in this study.
