# Supplementary material for: DNA sampling from eggshells and microsatellite genotyping in rare tropical birds: Case study on Brazilian Merganser
Source: Genet Mol Biol. 2017 Oct 2;40(4):808–12. doi: 10.1590/1678-4685-GMB-2016-0297 (PMC5738623; doi:10.1590/1678-4685-GMB-2016-0297)
Supplement: Supplementary file 1 [file 1415-4757-gmb-1678-4685-GMB-2016-0297-Suppl01.pdf]

Supplementary material to "DNA sampling from eggshells and microsatellite genotyping in rare tropical birds: case study on Brazilian Merganser"

Table S1. The 27 loci tested for amplification in this study.

Loci tested		Primers (5' - 3')	Repeat motif	Reference
Moc A11	F	CTGCCACTCCTTTGATCCTTT	$(TG)_9$	This study.
	R	ACAAGAGGGAGCAGCTTTCA		
Moc A12	F	CCAGGAGCTTCTCCAACAGGA	$(AC)_8$	This study.
	R	TTAATGGCATTTGGGGGTAA		
Moc B3	F	CTGAGCACTTTGCCCTGATAA	$(CA)_8$	This study.
	R	GGATTGCCCATTTCAGTGAT		
Moc B5	F	CGGAGAAATCCAAAGCTCCTG	$(AAT)_{5...}(TG)_8$	This study.
	R	GCTGGAAACACTTTGGCATC		
Moc C3	F	CAGGGCAACAAAATCAGGTTC	$(TG)_{10}$	This study.
	R	CCAAGTGAGAAACAAAACCAAA		
Moc C11	F	CTGGATGCATTGTTAGGAGAGG	$(GT)_8$GGTGT$(TG)_{10}$	This study.
	R	TCTTGCAGTGCATTTCTTGG		
Moc D4	F	CGCGCTAGCTGTAAGGCTCAT	$(AC)_8$	This study.
	R	CCAGAAAAGCCTGTGTTGGT		
Moc D5	F	CCTGCTCTGAATTGCTCCTAT	$(GT)_7$	This study.
	R	CTGCAGTAAATCAGTAAATCAC		
Moc D12	F	TCTCTTAGGCTTTTGGGTGGTG	$(AC)_9$	This study.
	R	GGCTTACGCGTGGACTAACT		
Moc E2	F	CTGTGCTTCAGTCACGTAGGG	$(TG)_8$	This study.
	R	ACCCAGGGATTTCAGTGATG		
Moc F9	F	CATTTTCCTCTGGGGCAGATT	$(AC)_9$	This study.
	R	TCTCAGAAGGACCACGAAGA		
Moc F12	F	CAAAGCTTCCTCCCTCACTCC	$(GT)_9$	This study.
	R	AGCTTCCTGTGGCATAGCAT		
Moc G3	F	CATATGGCATGGCATTGAAGG	$(GA)_{10}$	This study.
	R	CCGCACTAGTGATTCTCTTGC		
Moc G7	F	CGATTCCCTGCAGACTGGTTG	$(CA)_{13}$	This study.
	R	CCCCCTTCACAGAACCATAG		
Moc H3	F	CAGCTGCAGTCTGTGGGAGAT	$(CA)_2$TG$(CA)_7$	This study.
	R	CAGTGCAAAAGAGGGCAGAG		
Moc H5	F	CCAATGGGGTCATTGTTGAG	$(CA)_7$TACATA$(CA)_5$	This study.
	R	GCATTGTATTTACAGAGGCTAAAC		
Moc H7	F	CAAACAACACCTGGCACTGCT	$(TG)_8$	This study.
	R	AATTCCAGGCCTGCTGTTTA		
Moc H8	F	CTCTGGCTCTCCCAGACAACT	$(TG)_{18}(TA)_8$	This study.
	R	TGCATCCTTTCTCAGTAATTGG		
Aph 08	F	AAAGCCCTGTGAAGCGAGCTA	(CA)12	(Maak *et al.*, 2003)
	R	TGTGTGTGCATCTGGGTGTGT		
Aph 13	F	CAACGAGTGACAATGATAAAA	(GA)10	(Maak *et al.*, 2003)
	R	CAATGATCTCACTCCCAATAG		
Aph 15	F	TGAATATGCGTGGCTGAA	(CA)9	(Maak *et al.*, 2003)
	R	CAGTGAGGAATGTGTTTGAGTT		
Aph 20	F	ACCAGCCTAGCAAGCACTGT	(CA)9	(Maak *et al.*, 2003)
	R	GAGGCTTTAGGAGAGATTGAAAAA		

Loci tested		Primers (5’ - 3’)	Repeat motif	Reference
Hhi05	F	CTCTCCTTTTACTACAAATTCCCTT	(CA)13	(Buchholz *et al.,* 1998)
	R	ATAAAGGTAGGTGACCCAATCCT		
Mm 02	F	CCCTGCCCCATCTAACTAAAC	(CA)23	(Gautschi and Koller, 2005)
	R	GCTGCTTTGGTGGAGCAAC		
Mm 03	F	AAGTACATGTAAAAGCTGAAGTTGC	(CA)16	(Gautschi and Koller, 2005)
	R	TTGCCTGATAAAAGGAATGC		
Mm 04	F	CAGGCTCAATGAGGACAGG	(CA)16	(Gautschi and Koller, 2005)
	R	GCATCACCCTCCGTTTGG		
Mm 06	F	CACATGGAGGGTCAGCATC	(CA)22	
	R	CTGGGGGATGCAAGTCTG		(Gautschi and Koller, 2005)
